# Wayfinding in pairs: comparing the planning and navigation performance of dyads and individuals in a real-world environment

**DOI:** 10.1186/s41235-024-00563-9

**Published:** 2024-06-21

**Authors:** Crystal Bae, Daniel Montello, Mary Hegarty

**Affiliations:** 1 Department of Geography, University of California, Santa Barbara, Santa Barbara, CA USA; 2 Center for Spatial Data Science, The University of Chicago, Chicago, IL USA; 3 Department of Psychological and Brain Sciences, University of California, Santa Barbara, Santa Barbara, CA USA

**Keywords:** Social wayfinding, Navigation, Wayfinding, Social interaction, Route planning, Spatial cognition, Social decision-making

## Abstract

Navigation is essential to life, and it is cognitively complex, drawing on abilities such as prospective and situated planning, spatial memory, location recognition, and real-time decision-making. In many cases, day-to-day navigation is embedded in a social context where cognition and behavior are shaped by others, but the great majority of existing research in spatial cognition has focused on individuals. The two studies we report here contribute to our understanding of social wayfinding, assessing the performance of paired and individual navigators on a real-world wayfinding task in which they were instructed to minimize time and distance traveled. In the first study, we recruited 30 pairs of friends (familiar dyads); in the second, we recruited 30 solo participants (individuals). We compare the two studies to the results of an earlier study of 30 pairs of strangers (unfamiliar dyads). We draw out differences in performance with respect to spatial, social, and cognitive considerations. Of the three conditions, solo participants were least successful in reaching the destination accurately on their initial attempt. Friends traveled more efficiently than either strangers or individuals. Working with a partner also appeared to lend confidence to wayfinders: dyads of either familiarity type were more persistent than individuals in the navigation task, even after encountering challenges or making incorrect attempts. Route selection was additionally impacted by route complexity and unfamiliarity with the study area. Navigators explicitly used ease of remembering as a planning criterion, and the resulting differences in route complexity likely influenced success during enacted navigation.

**Supplementary Information:**

The online version contains supplementary material available at 10.1186/s41235-024-00563-9.

## Significance statement

Much of our daily wayfinding behavior is conducted with other people, yet limited research to date explicitly considers the social aspects of a task such as jointly planning and navigating a route. The present research makes a novel contribution to understanding how people work collaboratively to find their way together through a real-world environment. We detail important patterns of social and spatial behavior with regard to wayfinding in a realistic novel environment, with or without a partner. Results suggest that wayfinding as part of a dyad is fundamentally different from wayfinding as an individual. The social structure of a group itself, such as whether it consists of strangers or friends, may additionally impact wayfinding practices and success. Research in this area has implications for training people to navigate in groups, for the planning and design of the built environment, and for improving wayfinding aids. In terms of the implications for basic cognitive research, our studies expand our knowledge of decision-making within a realistic spatial context and with a social partner, drawing on the shared formation and recall of spatial route representations.

## Introduction

In our day-to-day lives, we often face the challenge of traveling through a new environment or navigating to a new location. Wayfinding is a process that comprises all of the cognitive and behavioral actions associated with planning the way between an origin and a destination, including recognizing landmarks, remembering routes, and orienting oneself within the environment. In most cases, wayfinding critically depends on our mental representations of physical environmental spaces, and sometimes external representations like maps. Navigation comprises wayfinding together with locomotion, which is the act of physically coordinating one’s body to move within the environment in question, to carry out the wayfinding task (Montello, 2005).

This daily navigation is also often social. In every day life, we commonly work as social collectives to perform the ubiquitous spatial tasks associated with spatial planning and navigational problem-solving (Montello et al., 2023). However, limited prior research investigates how navigation may work for pairs or groups of people: What strategies contribute to success in these types of interactions? What are some of the unique challenges facing pairs or groups of people in navigation? The present research is one of few works centrally focused on collaborative wayfinding in a real-world context (as advocated by Dalton et al., 2019). It builds on previous forays into the social dimensions of wayfinding (e.g. Burte, 2004; Daniel & Denis, 2004; He et al., 2015). For example, Burte (2004) compared individuals to dyads in planning routes and wayfinding in a real environment to find that individuals planned more quickly and recalled more items in a sketch map, executed routes more efficiently (in terms of both time and distance), and showed no difference between same-gender and mixed gender groups. Daniel and Denis (2004) examined social contexts in route direction-giving and found that working directly with social partners significantly increased the conciseness of the directions given by the group, as compared to those prepared by individuals working alone. Social wayfinding has also been examined in virtual settings, where the comparison of performance between individuals, dyads, and triads points to the different dynamics of group sizes on wayfinding (Brunyé et al., 2023).

Our research addresses several key theoretical issues in the study of wayfinding as a social or collective spatial task (an overview is provided by Montello et al., 2023). Collectivity can have a broad and impactful role in wayfinding, potentially influencing what people believe is true about the layout of the environment, what people attend to, how they reason, and how they respond emotionally and motivationally during wayfinding. The study we report here sheds light on the central issue of how dyads wayfind as compared to individuals, including whether dyads perform better or worse than individuals, and why. Among other things, this tells us something about how our understanding of solitary wayfinding applies to dyadic wayfinding or not.

Because we audio- and video-record both our dyads and our individual wayfinders during planning and travel, we can examine the interaction between dyad members and observable aspects of the process of reasoning during wayfinding. Since we give our dyads the task of intentionally wayfinding together while copresent, our task is an example of what Dalton et al. (2019) labeled “strong synchronous social wayfinding.” An important reasoning process during wayfinding is that of monitoring one’s orientation during travel, a case of metacognition. During our strong synchronous social wayfinding task, dyad members not only monitor their own orientation but likely that of the other dyad member; Fernández Velasco (2022) identified this as “collective metacognition.”

We know from research on social reasoning practices that people are better at critically evaluating others’ plans versus their own plans made in isolation (Mercier, 2016), and may thereby practice better planning and reasoning in a social wayfinding context. A study by He et al. (2015) on simultaneous route direction-giving and -receiving by pairs of participants indicated that pairs perform differently than individuals because of differences not only in their individual senses of direction but also in their interpersonal communication. They found that participants with a better sense of direction were better able to adjust to the needs of their partner, adjusting their navigation instructions accordingly. This demonstrates flexibility in social coordination between members of a dyad, which may partially overcome the disadvantages of poor individual ability. These studies taken together emphasize the effects of a real social setting on group planning and spatial discourse.

In our study, we consider how dyad members communicate with each other during planning and travel, and ask if some ways are more or less effective than others. We appraise how characteristics of dyads and of the individuals making up the dyad influence wayfinding reasoning, communication, and performance. For one, we specifically compare dyads of strangers to dyads of people who already know each other. And we ask how variations in personality and intellectual traits among individuals contribute to variation in the wayfinding of the dyad. For example, are there individual traits or ongoing interaction dynamics which help explain how the opinions of dyad members are ignored or attended to?

The paradigm used in the present studies has participants first plan a route to a goal location (prospective planning) and then attempt to follow that route in the environment (*in situ* navigation). The task of navigating in an environment based on memory of a planned route and on real-time planning is in line with prior research in wayfinding (Meilinger et al., 2008). Extant research also commonly imposes a time limit to the navigation task (Brunyé et al., 2017), as we do in our studies, which increases ecological validity in the applied paradigm. A series of experiments by Hölscher et al. (2011) supported a “profound difference” between prospective and situated planning, wherein participants modify their route-following *in situ*. Route planning as it plays out in situated navigation differs from prospective planning in that it is more incremental, akin to what Heft (1996) calls “a temporally unfolding interaction between the wayfinder and the affordances of the environment” (Hölscher et al., 2011, p. 244). This difference between situated and prospective wayfinding raises questions about physically and socially situated effects on wayfinding, as a collaborative task between pairs of people rather than a task performed by individuals for themselves or to provide route instructions for others.

### Our prior study with stranger dyads

The two studies we present here follow a previous study on route planning and navigation by stranger dyads (Bae & Montello, 2019). In this previous study, 30 pairs of participants who were previously unfamiliar to one another (stranger dyads) were recruited separately and introduced at the beginning of the study. Each dyad first worked together in a lab room to prospectively plan a fairly complex route through the environment between a specified origin and a destination point, using a paper map. Participants were then taken to the study area where they physically walked a route between those same points in the environment without the use of the paper map, relying only on memory and communication to complete the navigation. Dyads were assessed on their navigational performance, individual differences in sense of direction and personality, and adherence to their route plans. Both phases—planning and navigation—were video-recorded for the analysis of social interaction.

Results of this study showed wide variation in navigational performance between stranger dyads, although most of the dyads (26 of 30, or 87%) were successful in reaching the correct destination on their first attempt. We found that those who followed their originally reported plans, rather than attempted to develop a novel plan created *in situ*, traveled significantly more quickly and directly to their destination. We found that dyads traveled significantly further than they had planned (on average 34% longer), demonstrating differences in prospective and situated wayfinding, even when attempting to follow a previously devised route. Although a third of all stranger dyads (*N* = 10) in the study followed their route exactly as planned and reported, average overlap for all dyads between planned and enacted navigational routes was only 69.1% (*SD* = 32.4%). Explanations that dyads offered about deviations from their original plans were categorized as either *getting lost*, *taking a planned alternative*, or *taking a novel shortcut en route*. Individual differences in sense of direction scores and in personality factors, calculated either as averages or differences between dyad members’ scores, did not significantly account for performance. Being strangers at the start of the study, we assume the dyad members were not previously aware of their fellow member’s spatial abilities or personality, however.

Common challenges arose from dyads’ attempts to enact their original planned routes. Planning with explicit considerations for simplicity in one’s route appeared to relate to eventual success, even as participants who did so often selected cognitively simpler but longer routes. Navigation performance, measured as success in efficiently reaching the destination and minimizing time and distance, highlighted differences between situated and prospective planning. Participants needed to anticipate challenges in advance during planning, but often modified their route-following on the fly based on unexpected challenges. We also investigated strategies of social role-taking (leading and following) within dyads. Our results argued for further analysis of conversational interaction in collaborative wayfinding research. Although wayfinding performance was not reliably related to individual differences in sense of direction or personality with dyads of previous strangers, our further analyses of social interaction suggest wayfinding and communication differences that may contribute to navigational success as a dyad.

### Our present studies with friend dyads and with solo navigators

Results from our previous study on stranger dyads give us a baseline against which to compare the results for friend dyads and for solo navigators that we report here. In two studies, we examine the wayfinding processes of friend dyads and solo navigators because these are the most likely social scenarios in which we navigate on a daily basis—alone or with an acquaintance. Rarely do we find ourselves in natural situations navigating together with a complete stranger. By explicitly assessing the effects of prior social relationships, we ask how familiarity with one’s wayfinding partner impacts dyadic planning and navigation processes. Social interactional aspects relevant to navigation may be more pronounced in dyads with prior familiarity. And given that acquaintances are more likely to have prior beliefs about their partner’s characteristics, we may find stronger relationships of wayfinding success to abilities and personality.

Examining individuals performing the same task as dyads also allows us to draw comparisons between wayfinding dyads and solo wayfinders. Comparing the navigational performance of stranger dyads, friend dyads, and solo navigators together allows us to examine the role of social familiarity in the context of carrying out navigational routes. We further demonstrate the importance of considering route complexity during planning and outline the challenges that accompany performing map correspondence to the physical environment in a situation like we examine in our studies.

Importantly, we look at both prospective and situated planning, as prior work has shown meaningful differences in the planning activities of wayfinders when performed prospectively (ahead of time) versus when enacted in real-time in the physical, situated environment (Hölscher et al., 2011). In our studies, we present a task with strong ecological validity in terms of both the environmental and interpersonal context. We use an existing outdoor physical environment as our study site as well as including the important social component of wayfinding partners. Our solo navigator study serves as comparison to the dyad studies, highlighting the benefits and challenges introduced by collaboration with a partner, whether stranger or acquaintance.

As reviewed above, prior wayfinding research has mostly focused on solo wayfinding, leaving social aspects under-researched (Dalton et al., 2019). Spatial cognition research, in its search for empirical control, often ‘takes place’ in lab or virtual reality contexts, rather than in real-world settings. Virtual environments have been useful in the study individual spatial cognition (Coutrot et al., 2019; van der Ham et al., 2015), albeit with trade-offs in ecological validity. However, we suggest that ecological validity is further compromised in the case of social wayfinding. When trying to replicate realistic social interaction during navigation in a virtual context, it is more difficult in a virtual setting to study important natural features of communication with one’s partner, which is not limited to verbal speech but also modalities such as body language and gesture, coordinated alignment, and tracking of partner’s gaze. On the other end, there is also work in social wayfinding that uses highly realistic social contexts-more akin to ethnographic or naturalistic observation-with the trade-off of lower empirical control (Brown & Laurier, 2005). This work specifically investigates wayfinding in a real physical environment and in socially realistic contexts, increasing the ecological validity over lab-based spatial cognition research.

### Research questions

To assess the performance of friend dyads and solo wayfinders on route planning and navigation, and to compare across stranger dyads, friend dyads, and individuals, we pose the following research questions in these two studies (including their comparison to our previous study on stranger dyads):

- How do friend dyads work together in both navigational planning and situated wayfinding to reach a destination?
- How do solo wayfinders work to prospectively plan and carry out a situated navigation task?
- How do planning and navigation processes differ between stranger dyads, friend dyads, and solo wayfinders? What strategies or characteristics of individuals and groups account for navigational success and efficiency, whether navigating alone or with another person?

## General method

In both studies, participants used a cartographic map to plan a route between a given origin and destination point in a previously unfamiliar area, and then attempted to enact their route plans through physical navigation in the study area. Performance measures were (1) success in reaching the destination on the first attempt, (2) elapsed time, and (3) distance traveled. Activities and interactions were video- and audio-recorded during both the planning and navigation phases. For dyads, the recordings captured conversational dialogues, reflecting shifting attentional focus and planning strategies of the dyad; for solo navigators, who were instead asked to perform a think-aloud protocol throughout the same phases of the task, the recordings captured monologues that reflected shifts in solo attentional focus and planning strategies. In addition, individual difference measures of sense of direction and personality were administered.

### Materials and study site

**Fig. 1:**
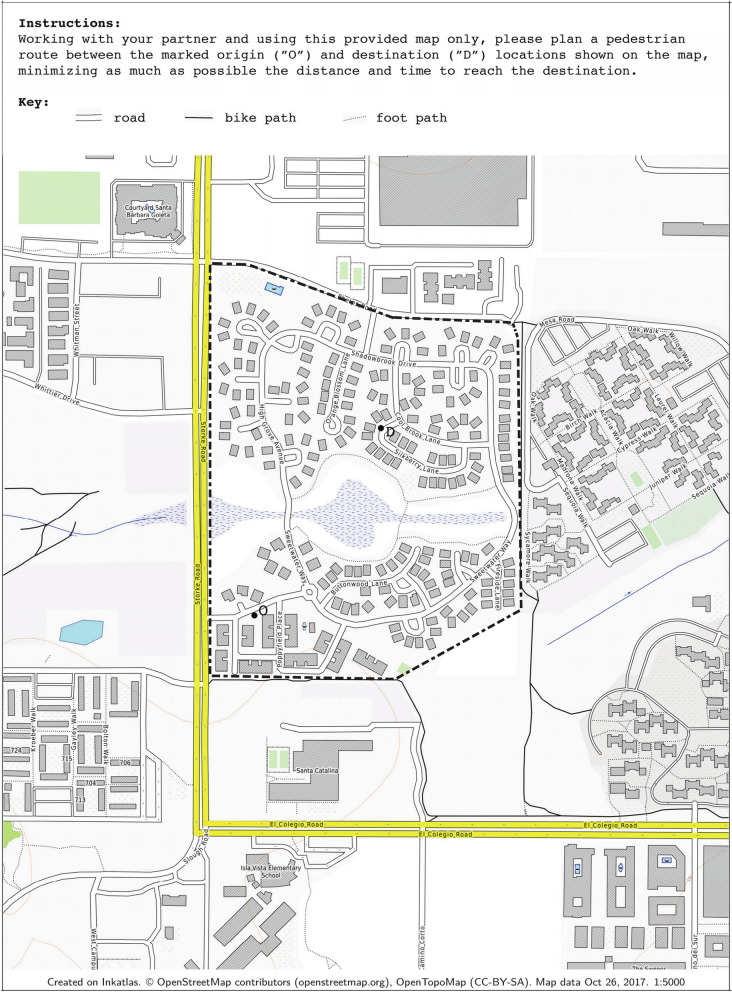
Paper map used by participants for route planning

Each study took place in a university lab space for the planning phase and in a residential suburban neighborhood approximately 1.5 miles away for the navigation phase. The study site was selected because the layout is complex enough to likely pose a moderate level of wayfinding challenge for most people. The neighborhood has a largely curvilinear street structure, pedestrian-friendly and low-traffic streets, cul-de-sacs branching off of the main access, and a central open space with interior footpaths (see Fig. 1). There is little elevation change throughout, so there were no locations providing visual access to the entire layout.

Participants completed online questionnaires in advance of study participation: the Santa Barbara Sense of Direction scale (SBSOD; Hegarty et al., 2002) and the Big Five personality inventory (John et al., 2008). The SBSOD is a generalized self-report measure of navigation ability, commonly used in spatial cognition research. The personality inventory used here is based on the five-factor model of personality, referred to as the “Big Five” structure of personality consisting of the main factors of *Extraversion*, *Agreeableness*, *Conscientiousness*, *Neuroticism*, and *Openness to Experience*.

For the route planning phase, participants planned their route from the location marked “O” (origin) to the destination marked “D” (destination) using the paper map shown in Fig. 1. The map instructions were read to the participants and also printed on the map itself: “Working with your partner and using this provided map only, please plan a pedestrian route between the marked origin (“O”) and destination (“D”) locations shown on this map, minimizing as much as possible the distance and time to reach the destination.” Throughout the study, participants were video-recorded with a camcorder mounted on a tripod in the lab room, and with both a handheld camcorder (held by the researcher) and a chest-mounted GoPro action sports camera (worn by participants) in the neighborhood study environment. The video cameras were visible to participants, who were informed about each phase that would be recorded. Participants were GPS-tracked by the researcher in the navigation phase using a cell phone application.

### Procedure

Participants were recruited online through a departmental research pool which included undergraduate students across a variety of majors.^1^ Both members of each dyad (in Study 1) or the individual participant (in Study 2) met the researcher at the lab for initial questionnaires and were asked to plan a pedestrian route between the given origin and destination locations marked on the map in a neighborhood near campus. Before planning, participants were informed that they would be taken in-person to the neighborhood in the next phase of the study to navigate to their destination *without* the use of the map. They were given up to 10 min for planning. Immediately after planning, each participant was asked to draw and describe their route plan to the researcher in a separate room, to confirm the main planned route, and for Study 1, the agreement between dyad members.

Following the planning phase, participants were driven to the origin point within the study site and instructed to physically navigate to the destination, minimizing time and distance traveled. Participants were allowed to modify their route plan as needed when navigating *in situ*. The researcher followed the participants during navigation to record a GPS track of the travel route, note elapsed time for the navigation phase, and keep track of “attempts” made, where the participants notified the researcher that they believed they had reached the destination (whether correct or incorrect). Participants were given up to 3 attempts to reach the destination, and were not required to return to the origin point between attempts.

The navigation phase ended when the participant(s) either reached and identified the destination successfully, identified an incorrect destination location on three attempts, exceeded the maximum time limit of 30 min, or gave up, whichever came first. An attempt was counted when both members of the dyad or the individual (in the case of solo wayfinders) verbally identified to the researcher that they believed they were standing at the destination. There was no salient landmark marking the destination, so participants had to identify it based on the remembered location from the map. The researcher then informed participants whether they had correctly identified the destination, and if not, how many attempts were remaining. Those who were incorrect but had remaining attempts and time were allowed to continue from their present location.

Afterward, the researcher walked the participant(s) to a nearby location within the study neighborhood where each member individually completed the follow-up questionnaire before being debriefed and taken back to campus. The follow-up post-navigation questionnaire further clarified participants’ assessments of their performance during the navigation, including whether they made any deviations from the planned route (see Post-Navigation Survey in the Supplemental Materials).

### Data processing and scoring

#### Analysis of planning

To assess the routes planned by participants, the routes drawn on paper maps and reported by participants in the video recordings during the planning phase were characterized and coded by the researchers. Data were recorded on which route each individual member of the participant dyad (or solo participant) reported. Both the route drawings on the paper map and the video recordings were used solely to code which route was selected and reported by the participants, rather than assess in detail the conversation around the planning process.

**Fig. 2:**
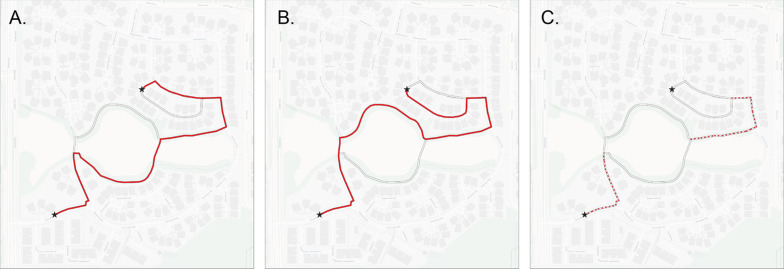
**A** First example route as digitized from drawn route plans and represented as a red line.** B** Second example route in red.** C** Overlapping segments in A and B, displayed as a dotted red line

Because routes were drawn by participants on paper maps, the maps were scanned and geo-referenced in Geographic Information Systems (GIS) software, where routes were manually digitized by the researcher to determine the path of the planned navigational route. Drawn routes were snapped to nearest road or path vertices based on the underlying network representation. See Fig. 2A, B to see examples of two digitized route plans. Additional metrics could then be calculated from the digitized route, such as length of the planned route and overlap between routes, such as demonstrated in Fig. 2C as the overlapping segments of the routes in A and B.

Video-recordings of the individually reported routes—one for each participant—were used to verify the route drawn and determine agreement between participants on the planned route. Each “unique” route was based on visibly distinguishable sequences of segments, and was given a letter code labeling it to identify and code participants’ planned routes.

#### Analysis of navigation

For processing data from the navigation phase, the recorded GPS tracks were used in conjunction with the video recordings from all cameras to verify the route taken by each dyad or individual. Travel time for navigation (including any pausing during travel) was noted by the researcher at the end of the navigation phase, and GPS recordings were additionally used as a verification of time elapsed during travel. Participants were scored as successful in reaching the destination if they traveled to and identified the correct destination location on their first attempt, within the 30 min time limit. First attempt was defined as the first location claimed as the destination, whether or not it was correctly identified. For all participants who ultimately reached the destination within the 3 allotted attempts and within the time limit, the number of attempts required to do so were noted by the researcher.

For the enacted routes, the GPS trajectories from navigation were imported into GIS software and snapped to vertices based on the network representation as before to account for GPS receiver noise. For comparison between planned and enacted routes, overlapping segments between the GIS representation of the enacted and planned routes were extracted using the ArcGIS Intersect tool. The visual representation of this matches what is shown in Fig. 2.

For our qualitative analysis of the social interaction during navigation, we apply the Conversation Analysis framework in our assessment of how dyads coordinate their plans during travel. A key feature of the Conversation Analytic approach to data collection (Goodwin and Heritage, 1990; Schegloff, 1991) is its concern with the study of talk-in-interaction, naturally occurring conversation as it unfolds within a socially-shared context. This is done through the close analysis of video recorded data of everyday interaction. The use of the Conversation Analysis framework is appropriate to apply in the cases of situated social wayfinding, as it recognizes the multimodality of communication through methods of transcription and coding of both speech and gesture.

To analyze the video recordings from the navigation phase, we first transcribed speech and gesture by participants from the videos using the open source transcription software ELAN (Wittenburg et al., 2006). We follow transcription conventions as outlined in Conversation Analysis (Sacks et al., 1974), which is seen in the analyses of conversations and think-aloud protocols in our results. We similarly transcribed and coded individuals’ speech and gesture for analysis of the think-aloud protocol.

## Study 1: Friend dyads

Study 1 investigates coordinated spatial wayfinding as prospectively planned and as it occurs *in-situ* by dyads who are formerly acquainted and in an established social relationship with one another (referred to as “friend dyads”). It differs from our previous study with stranger dyads (Bae & Montello, 2019) by following participants in a more socially realistic scenario, in which partners are known to one another, exposing the influence of prior relationships between the members of each pair. Participants in this study comprised a new set of 30 dyads, each of whom self-identified as friends, performing the same task as in our previous stranger dyads study and following the same procedure, as detailed in the previous section.

We hypothesized that social interactive aspects relevant to navigation would be more pronounced in dyads with prior familiarity, such as familiar dyads exhibiting more pronounced social role-taking or more communication efficiency (such as faster planning) between partners. Members of dyads who have an established social relationship with one another are likely to have greater preconceived notions of each other’s relative spatial navigation ability and personality. Members of such dyads are also more likely to understand and hold expectations of each other’s communication styles, social roles, and overall decision-making processes. They may additionally more readily and comfortably enact either leader-follower or collaborative social roles within their dyad, so we may see clearer expressions of leadership.

### Participants

Thirty pairs of participants were recruited for this study, none of whom had participated in the previous study reported in Bae and Montello (2019). At least one member of each dyad was an undergraduate student who signed up through the research pool.^2^ For each participant dyad, the member who registered in the study freely selected a friend to participate with them at time of sign-up. Age of participants ranged from 18 to 25 years old (*M* = 19.1, *SD* = 1.4).

A summary of individual difference measures for all participants (*n* = 60) across the 30 dyads in the study is given below in Table 1. None of the measures significantly varied by gender in this study, as reported in the “Gender Differences” column in the table. Additionally, participants in this study did not significantly differ from those in the previous study on these individual difference measures; comparisons of individual differences across all studies are reported in the Study 2 results and in Table 7.

**Table 1:** Means on SBSOD and Big Five Inventory for participants (*n* = 60) in Study 1

Measures	All members [range]	Female (*n* = 40)	Male (*n* = 20)	Gender differences (all not significant)
SBSOD	4.0 [2.0–6.6]	3.9	4.4	*t*(33.37) = − 1.62, *p* = .12
Extraversion	3.4 [1.4–5.0]	3.5	3.2	*t*(43.35) = 1.51, *p* = .14
Agreeableness	3.8 [2.6–4.9]	3.9	3.7	*t*(38.27) = 1.22, *p* = .23
Conscientiousness	3.4 [1.8–5.0]	3.5	3.2	*t*(39.16) = 1.77, *p* = .08
Neuroticism	3.0 [1.1–5.0]	3.1	2.8	*t*(33.10) = 1.67, *p* = .10
Openness	3.4 [2.3–5.0]	3.4	3.4	*t*(32.91) = 0.34, *p* = .74

Length of friendship in each dyad and prior familiarity of members of the dyad were assessed by self-report at the start of each session. For the purposes of this study, dyads were verified to have a prior social relationship if they had known one another for at least a year (12 months) and mutually rated each other either “friends,” “best friends,” or “romantic partners,” as opposed to only “acquaintances,” “classmates,” or “those who spent occasional time together.” The average length of friendship across all 30 dyads was 3.3 years (*SD* = 3.1 years).

The gender pairings in the study consisted of 13 female–female (F-F) dyads representing 43.3% of the sample, 14 female-male (F-M) dyads representing 46.7% of the sample, and 3 male-male (M-M) dyads representing 10% of the sample. This relative lack of M-M dyads was similar to our previous study with stranger dyads^3^; it reflects the gender composition of the volunteer pool. When the study site was described and shown to participants on an overview map, all 60 participants claimed to be either “very unfamiliar” (*n* = 54) or “unfamiliar” (*n* = 6) with the study environment, as measured on a 5-point scale ranging from “very unfamiliar” to “highly familiar.” Prior familiarity with the environment is thus not a factor in performance of the task.

### Results

#### Route planning by friend dyads

The average planning time for friend dyads was 2 min 59 s, and this did not vary between those who were successful during navigation and those who were not.

Overall, the 30 pairs of friends proposed and reported 16 unique route plans. It is notable that so many creative plans were proposed, planned, and reported for a map and environment that appears otherwise constrained in its solution space. For instance, several of these route plans decide in advance to stray off the labeled paths and roads; see Fig. 7 for overlaid examples of unique route plans. During the planning process, friend dyads also demonstrated consideration of alternative routes, rather than a commitment or explicit agreement on only one main route plan. We compared the success of those friend dyads who considered multiple plans vs. just one and found no significant difference in their eventual navigational success, $\chi ^2$ (2, *N* = 30) = 0.83, *p* = .36.

#### Navigational performance by friend dyads

We used success in reaching the destination, navigation time, and navigation distance as measures of navigational performance. As in the stranger dyads study, any friend dyad who correctly identified the destination on their first attempt was considered successful.

In total, 22 of 30 friend dyads (73%) reached the destination correctly on their first attempt and were considered successful in the task. Of those 8 friend dyads who were unsuccessful, 3 reached the destination on their second attempt, and the other 5 dyads were unable to reach the destination within their 3 allotted attempts. No friend dyads were stopped due to exceeding the 30 min time limit. Overall total time navigating for all 30 friend dyads averaged 9′ 14″ (9 min and 14 s), with a range from the shortest navigation time of 5′ 25″ to the longest time taken, 21′ 30″, and a standard deviation of 3′ 49″. Total navigation time was highly correlated with distance traveled, *r* = .86, *p* <.001, for friend dyads. Overall total distance averaged 0.52 miles, ranging from a distance of 0.36 miles to 0.92 miles, with a standard deviation of 0.13 miles.

#### Correspondence between planned and enacted routes for friend dyads

Next, we characterize how closely participants followed those routes they planned in advance when they were navigating within the environment. In this context, we are using the term *correspondence* to refer to the relationship between the reported prospective route plan and the enacted navigational route in the environment. Correspondence is related to the act of translating between the two-dimensional physical or internal map representation to the experienced physical environment. Assessing correspondence allows us to characterize where participants either took intentional alternatives to their original route plans or where they got off course, knowingly or not. To compare distance traveled in the environment to the distance of the planned route, we report the *distance ratio* as:

$$\begin{aligned} \text {Distance Ratio} = \dfrac{\text {Distance of Enacted Route}}{\text {Distance of Planned Route}} \end{aligned}$$

This ratio equals 1.0 if the enacted and planned routes match in distance, although that would not necessarily indicate enactment of the same route. The distance ratio between the length of the route enacted during navigation and the length of the planned route(s) averaged 1.22 for pairs of friends. This is significantly different from a distance ratio of 1.0, *t*(59) = 3.4, *p* <.005, meaning dyads traveled further than planned.

To more precisely consider the overlap between the planned route and the enacted route, we also calculate adherence to the specific planned route during navigation as a measure of *route overlap*:

$$\begin{aligned} \text {Route Overlap} = \dfrac{\text {Distance of Overlapping Segments}}{\text {Distance of Enacted Route}} \end{aligned}$$

See the above Sect. 2.3 for information on the calculation of overlapping segments. When the planned route exactly matches the enacted route, this route overlap ratio equals 1.0 (100%). For friend dyads, average route overlap was 75.4% (*SD* = 31.0%) and ranged from 100.0 to 17.1%.

There was a significant negative correlation between dyad members’ averaged SBSOD scores and the degree of route overlap (*r* = − 0.39, *p* <.05), meaning those friend dyads with better SOD followed their planned routes less closely. Over half of the friend dyads (16 of 30) followed their route exactly as planned, with 100% overlap. As expected, those who more closely followed their reported plans took less time (*r* = − 0.65, *p* <.05) and traveled a shorter distance (*r* = − 0.31, *p* <.001) to the first attempted destination.

#### Conversational analysis for friend dyads

From the social interactive analysis for the friend dyads, we note several recurring types of responses to deal with these memory concerns: planning routes that are easier to remember; simplifying individual and shared mental representations of the route plan; and subdividing the task of remembering the route between the two dyad members. To provide an illustrative example, we find in the analysis of video recordings that members of friend dyads more often explicitly raised uncertainty about the “possibility” of carrying out suggested routes than did stranger dyads in the previous work.

**Fig. 3:**
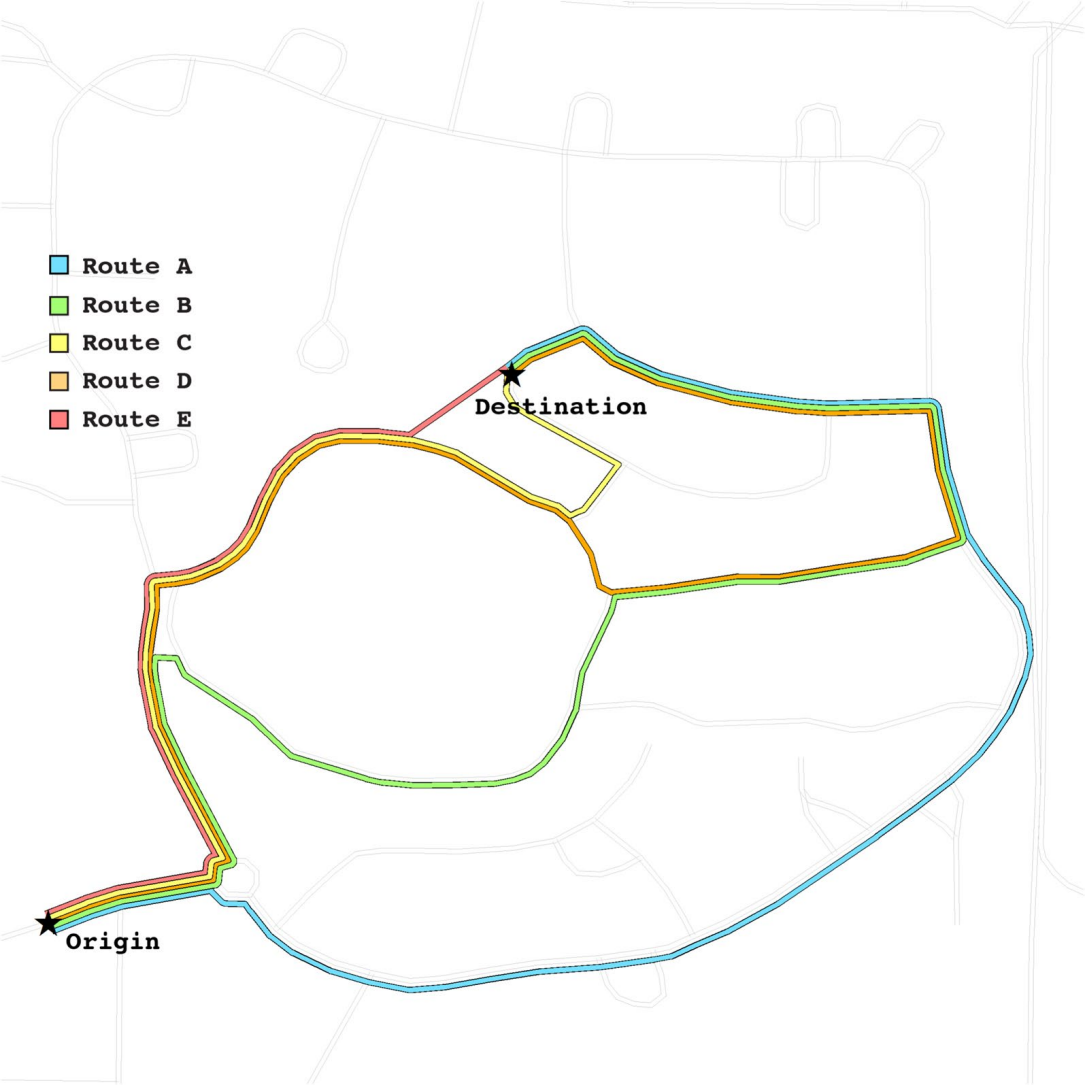
Five most popular route plans across all three studies

The following characterization quotes from one friend dyad’s planning process, where the speakers are aligned to the risk involved with failing to carry out a route successfully. To directly discuss the justification for planning a simpler route, one of the dyad members begins, “I feel like for us specifically...” then trails off and pauses as their partner traces a route with their finger that follows the main road around to the destination. The plan in question is the most common across all study participants, shown as Plan A in Fig. 3. The first speaker confirms by responding: “yeah just taking the road,” showing that they agree on the suggested ‘simpler’ plan. Immediately following this the speaker states, “I don’t know if I trust us enough to just cut like straight through.” Here they allude to their shared (in)ability to remember and carry out an alternate road which uses the footpaths rather than the longer way around staying on the simpler main perimeter road. Many dyads in this way decided upon what they believed to be an “easier to remember” route among multiple options, perhaps to purposefully preempt potential issues of memory.

Other examples from the recordings show that when one partner suggests cutting through an area not explicitly marked as a path on the map, the dyad commonly and pre-emptively considers alternatives to take in case the plan turns out to be impossible in the situated navigation. This suggests either that friends may be better able to imagine or visualize future problems during planning, or more likely that they are more likely to raise such issues in communication than relative strangers.

In addition to these examples of social coordination during planning, friend dyads commonly collaborated to jointly recall their route and worked together to diagnose navigational issues such as recognizing relevant landmarks during enacted navigation. In this next excerpt, we see examples of both^4^:
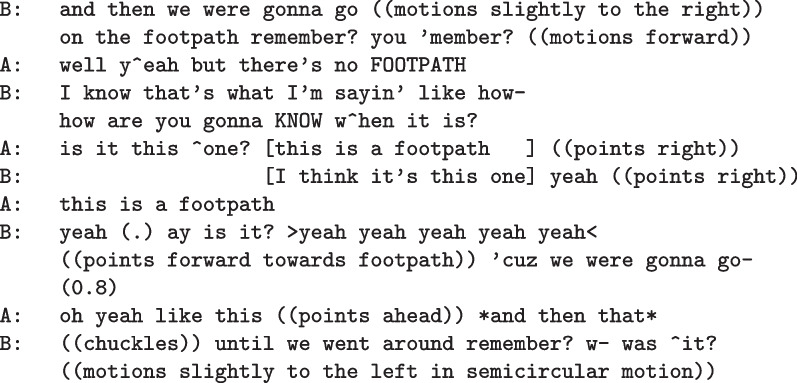


Not seeing anything that corresponds yet to what they would recognize as a footpath, speaker B asks how they will be able to “know when it is.” This both suggests the footpath is further away from the last turn than they may have expected and jointly draws their attention to the active search for the start of the footpath. Once they recognize the sidewalk leading into the inner area as a candidate for the footpath, they each identify the footpath nearly in overlapping talk. Speaker A first points to the right and asks “is it this one?” as their partner quickly follows with a similar gesture and a hedged confirmation, “I think it’s this one.” Speaker B asserts this more strongly as they move closer and gain visibility of the start of the footpath. They then continue to rehearse their planned route based on their current navigational progress.

This demonstrates the challenges of identifying specific landmarks, based both on visual identification of the footpath and primed through anticipation of where such a landmark should occur along their progress within the environment, and additionally shows how friend dyads use social resources to coordinate their action and planning. By drawing their joint attention to the task of identifying the landmark, this dyad accurately achieves this slightly ahead of where they can fully view the footpath and is able to do so without slowing down or pausing their progress toward their goal.

### Discussion

This study furthers our understanding of collaborative planning and navigation in an environment with high ecological validity, both in terms of the task environment and the social scenario, by building on previous work with stranger dyads. Friend dyads spent about as much time as strangers to prospectively plan their routes, but proposed more unique route plans and traveled more efficiently together in the situated navigation task. Friends’ close adherence to their plans and subsequent shorter travel suggest the importance of planning to efficient wayfinding. However, efficiency did not always result in wayfinding success, as fewer friend dyads were successful overall in reaching the correct destination. Social familiarity within the dyad may have lent friends more wayfinding confidence, which could both have resulted in efficient navigation and may have also led them to be less flexible in adapting their plans in the situated context due to over-confidence.

Therefore, we believe that friends’ navigation is more efficient in part because friends consider more alternative routes ahead of time, giving them more options for changing course en route. Although this can allow for better adaptation to the environment, it can also enable overconfident behavior, possibly leading to exploring more uncertain shortcuts. It seems that working with a familiar social partner could inspire more confidence and enable a certain amount of risk-taking in a wayfinding scenario, as compared to working with a stranger.

To this point, we did also note a relationship between higher average dyad SBSOD scores and less route overlap. Friend dyads with higher SOD scores may have been more likely to try alternatives to their original plans, which increased their chances of becoming lost en route despite the social resources available to them as seen in the analysis of their conversations.

## Study 2: Solo wayfinders

Having examined wayfinding by friend dyads as a comparison to the wayfinding by stranger dyads in Bae and Montello (2019), in Study 2 we compare dyadic wayfinding to wayfinding by individuals working alone. This allows us to outline better the contributions of social aspects of wayfinding to performance. To do this, we recruited individual participants to perform a task similar to that of the dyads in the previous studies. Examining solo navigation allows us to highlight the benefits or challenges introduced by collaborating with a partner.

The main questions we explore in this study are whether solo navigators’ wayfinding performance differs from that of dyads on a comparable planning and navigation task, and if so, to what extent individual differences and interactive strategies within dyads contribute to performance differences. Of course, individuals generally do not verbalize their thought processes during navigation the way that members of dyads and groups necessarily do to communicate among themselves, especially when asked to work together on a task. In order to make the individual and dyadic conditions more similar in this respect, we had individual navigators carry out a video-recorded think-aloud protocol (Ericsson & Simon, 1980) while they traveled their route. This provides an analog to the conversations dyads have during travel, and secondarily gives us a source of information about individual’s decision-making processes during wayfinding. It also avoids a confound by making participants aware they were being video recorded in both dyad and individual studies.

Think-aloud protocols have previously been employed in wayfinding studies, typically to elicit spatial considerations during decision-making. For example, Passini (1992) used think-aloud in a set of wayfinding studies to inform architecture and design, describing in detail the methodology for having participants verbalize their decision-making in multi-level indoor and urban outdoor spaces. Raubal et al. (1997) also explored peoples’ verbal descriptions by interviewing them about their imagined wayfinding while looking at photos of sequential locations in the complex indoor space of an airport, connecting the analysis of these verbal descriptions to spatial image schemata. Broadly speaking, the usage of the think-aloud protocol allows us to study how individuals’ cognition as a sole wayfinder differs from the social cognition of a dyad performing the same wayfinding task.

### Participants

Thirty individuals were recruited from the same departmental research pool to participate in this study, none of whom had participated in either of the previous two dyad studies. The age of the 30 participants, 17 of whom identified as female and 13 as male, ranged from 18 to 25 years old (*M* = 20.7, *SD* = 2.1). Individual difference measures did not significantly differ between females and males in this study, as shown in Table 2. All 30 participants claimed to be either “very unfamiliar” (*n* = 23), “unfamiliar” (*n* = 5), or “somewhat familiar” (*n* = 2) with the study environment. Both participants who reported being “somewhat familiar” said they had been inside the neighborhood to visit a residence on only a single occasion. Thus, prior familiarity with the environment was likely not a factor in planning nor wayfinding performance.

**Table 2:** Means on SBSOD and Big Five Inventory for solo participants (*n* = 30) in Study 2

Measures	All members [range]	Female (*n* = 17)	Male (*n* = 13)	Gender differences (all not significant)
SBSOD	3.9 [1.1–5.7]	4.2	3.5	*t*(27.58) = 1.59, *p* = .12
Extraversion	3.2 [1.5–5.0]	3.4	2.9	*t*(23.88) = 1.36, *p* = .19
Agreeableness	3.8 [2.0–5.0]	4.0	3.7	*t*(23.37) = 1.33, *p* = .20
Conscientiousness	3.7 [2.2–5.0]	3.7	3.7	*t*(27.67) = 0.09, *p* = .93
Neuroticism	2.8 [1.0–4.4]	2.8	2.7	*t*(27.33) = 0.27, *p* = .79
Openness	3.5 [2.4–4.6]	3.6	3.4	*t*(26.75) = 0.71, *p* = .49

Individual difference measures of sense of direction and personality were similar to those of participants in the previous two studies; see “Table 7 in Appendix 1” for means of individual difference measures by study, as well as one-way ANOVA results demonstrating that means did not significantly differ across the participants in the three studies.

### Results

#### Solo route planning

Time spent by solo wayfinders to plan their routes in the lab ranged from 0′ 40″ to 10′ 40″ with an average planning time of 2′ 55″. Planning time was not associated with success, as measured both by those who reached the destination on their first attempt and by time and distance measures. Solo wayfinders reported only 9 unique route plans, comparable to the 9 plans for stranger dyads, but fewer than the 16 different plans reported for friend dyads.

However, after reporting their primary route plan, participants were further asked whether they considered any alternative routes during their planning and the majority reported doing so. The addition of this question was inspired by unprompted discussions of contingency (‘alternate’ or ‘back-up’) route plans by friend dyads in the prior study. In 90% of cases (27 of 30 individuals), solo participants reported at least one alternative to the reported route plan when asked. Although some provided descriptions of up to four or more alternate routes considered, participants were not prompted to explain more than one alternative route in response to this question.

#### Solo navigational performance

Of the 30 solo participants in this study, 16 individuals (53%) were considered successful because they reached the destination on the first attempt.^5^ Of the other 14 individuals, 7 of them did eventually reach and identify the correct destination location on their second or third attempt. Of the other 7 unsuccessful individuals, 4 gave up before making it to the destination, 2 were incorrect on all three attempts, and 1 was stopped because they ran out of time. Overall time for navigation averaged 12′ 22″ (12 min and 22 s) with a range of 5′ 50″ to 30′ 00″ and standard deviation of 6′ 26″. Overall navigation distance averaged 0.70 miles, with a range of 0.36 to 1.76 miles and standard deviation of 0.33 miles.

In analyzing navigational efficiency to the first attempted destination, we excluded the one participant who did not reach and identify a possible destination location before running out of time (30 min). Solo navigators’ time and distance on their first attempt—which was correct for those who succeeded and the first incorrect guess for those who did not—averaged 10′ 07″ and 0.58 miles.

The individual differences measures (SBSOD and personality) did not relate much to navigation performance. Sense of direction was not significantly correlated with wayfinding success, neither based on time to first attempt, *r* = 0.16, *p* = .41; nor distance to first attempt, *r* = 0.17, *p* = .38. Only one of the personality dimensions, Openness to New Experience, related to wayfinding performance. Higher scores on Openness to New Experience correlated with less efficient travel, i.e. *greater* time and distance traveled to the first attempted destination (*r* = .48, *p* <.01, with time; *r* = .42, *p* <.05, with distance).

#### Correspondence between planned and enacted routes for solo navigators

As before, to assess the correspondence between planned and enacted routes, we calculated both the *distance ratio* and the *route overlap* as measures of participants’ adherence to their original route plans. For individuals in this study, the distance ratio ranged from 0.64 to 5.07. Three participants traveled a shorter distance than their reported plan (distance ratio below 1.0). Most solo navigators (19 of 30) traveled farther than intended, with one person who walked more than 5 times the distance of their original route plan. The 30 individuals in this study averaged a distance ratio of 1.55.

Overall route overlap for solo navigators averaged 65.3%. The correlations between route overlap and time and distance to the (first attempted) destination were negative; *r* = − .47, *p* = .011 for distance and *r* = − .60, *p* <.001 for time. Therefore, when there was more overlap between the planned and enacted routes, solo navigators traveled faster to the first attempted destination.

Eight of the solo participants followed their route exactly as planned without deviation from the reported plan. Thus, it appears that more solo participants either got lost or took alternate routes from their primary planned route, as compared to the dyads in the previous studies. This is corroborated by the solo participants’ responses to the post-navigation question about whether and why they deviated from their planned route. Twelve of the 30 solo navigators (40%) reported following the same route they had planned, although only 8 of them had enacted it exactly. Of the 18 individuals (60%) who reported they did not take their originally planned route, 14 of the reasons were coded as “lost”, 2 as “alternate”, and 2 as “shortcut”. Thus, participants frequently took a route that they recognized as a deviation from their original plan (or plans), and in the majority of those cases, the deviation resulted from becoming lost while navigating.

#### Think-aloud protocol analysis

To further investigate strategies and challenges in individual wayfinding, the think-aloud protocol in our study elicits the main features of solo participants’ planning as well as their strategies for implementing their route plan *in situ*. Common topics of the think-aloud protocol during planning mirrored the topics of conversation in the dyadic planning process. These included identifying main map features, route comparison and selection, and verbal route rehearsal with simplification of the plan. Solo navigators spoke minimally and appeared to be doing much of the route comparison stage of planning silently. In some cases, individuals only started speaking their thoughts aloud to verbalize a route plan after first looking at the map silently for a period of time.

During the enacted navigation, the majority of topics verbalized included: rehearsal of plans, anticipation of upcoming decision points and their associated actions (turns or continuations), and adapting to unexpected circumstances such as changing one’s plan en-route. Solo participants rehearsed plans in much the same way as dyads did in paired communication, by rehearsing the relevant steps as encoded during planning. However, solo navigators often did so in a more halting fashion, with no conversational partner to help rehearse the plan more fluidly or otherwise fill in the pauses.

In many cases, individuals spoke not only about their thoughts or planning actions, but their navigational behavior as well. It was common for participants to narrate their actions as they were making them, e.g. “I’m making a left onto [Street Name]” or “keep going straight”. In the wayfinding context, these are still considered relevant to the think-aloud protocol, as it was overwhelmingly more common to narrate an action prior to taking the action rather than concurrent with it. This echoes findings by Brunyé et al. (2018), which show that the process of decision-making begins well before reaching a relevant intersection during wayfinding.

The think-aloud protocol analysis also reveals individual expressions of uncertainty. There was a considerable amount of self-reflection on one’s spatial or memory abilities, especially in relation to one’s own progress—such as when believing oneself to be possibly lost. Solo participants additionally expressed uncertainty about *whether* one was lost, as it was often unclear to participants whether they had gone off-course or were still on track to their destination as planned. Not having a wayfinding partner in these cases seemed to give individuals few resources for dealing with the challenges of disorientation or misremembering.

Individuals frequently hesitated to take risks, and may have had lower thresholds for tolerating uncertainty than dyads. Not only did some solo participants give up entirely on finding their destination (as stated above), but some also decided against attempting potential shortcuts. For instance, one individual who considered shortcutting through the middle area using footpaths during planning decides against it when approaching the possibility during situated navigation:
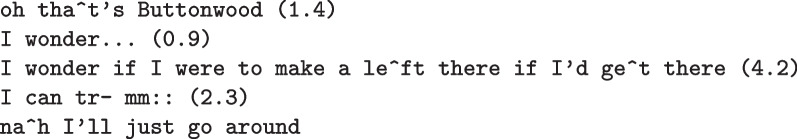


Examining video recordings of individuals’ planning and navigation processes demonstrates the value of having another method of understanding the cognitive processes during planning and navigation, but also illustrates the challenges in doing so with a think-aloud protocol. This examination points to planning strategies employed such as considering multiple routes, as well as specific challenges during the situated wayfinding task, such as the specific locations where uncertainty arises for an individual, mentions of what is not remembered (e.g. the relevant street names), and decisions made during the course of navigation.

### Discussion

In Study 2, we found that solo navigators performed fairly poorly on the wayfinding task, with only 16 of 30 reaching the destination on their first attempt. Four (13%) of the solo navigators who failed on their first attempt also gave up before making all attempts, without having exhausted their attempts or time. Forfeiting behavior was not previously discussed because it was not observed among dyads in either of the previous studies, making it notable to mention for the individual case. Again, as was the case with strangers and with friends, solo navigators who were successful in reaching the destination on their first attempt traveled more efficiently to their first (and only) attempt than did those who did not succeed on their first attempt; this appears to reflect confidence in their ongoing navigational progress, for those successful.

Differences in efficiency during navigation were not attributable to differences in sense of direction or personality characteristics other than Openness to New Experience, which was negatively correlated with efficiency. Bryant’s 1982 study of sense of direction and personality, although using different measures, similarly showed only a few personality correlates (namely, Flexibility). In that study, those with a lower Flexibility, interpreted as a greater need to stick to a plan or routine, had a higher SOD (Bryant, 1982); in our study with solo navigators we see that lower Openness to New Experience correlated with better navigation performance. This appears consistent with the interpretation that individuals with higher Openness may have more confidence in planning complex routes or trying shortcuts during navigation, which may mislead them. Openness to New Experience may characterize individuals who select more difficult routes in the first place (such as planning a route incorporating the footpath). These individuals may also have been more inclined to try making an unplanned turn to reach the destination faster, even when not part of their original plan.

The use of the think-aloud protocol for this comparison of planning and navigation gives additional insight into individuals’ planning, reasoning, and decision-making. Common topics in the think-aloud protocol demonstrate how anticipated and current wayfinding challenges are considered by individuals. These individual think-aloud protocols support our claims, especially the claim that more successful participants planned and navigated adaptively.

## Comparisons of performance across the three studies

Next, we make general comparisons drawing from the results of both studies presented here (Study 1 for friend dyads; Study 2 for solo wayfinders) as well as the previously reported study of stranger dyads in Bae and Montello (2019).

### Comparison of planning

We first compare the planning process across all three studies through the reporting of average time taken during the planning phase and average length of the routes planned and reported. Table 3 reports average planning time and average length of routes planned for stranger dyads in the previously reported study, for friend dyads in Study 1, and for solo wayfinders in Study 2.

**Table 3:** Comparison of planning across the three studies

	Avg. Planning Time	Avg. length of route plan
Previous Study (Stranger Dyads)	3′ 25″	0.50 miles
Study 1 (Friend Dyads)	2′ 59″	0.45 miles
Study 2 (Solo Wayfinders)	2′ 55″	0.49 miles

As noted in the table, planning times were fairly similar across studies—averaging 3 min total of the allotted 10 min for planning. It appears that stranger dyads took the longest to plan, but this was only a numerical difference rather than a statistically reliable one. The average length of the main route planned by participants in all three studies appear to be about the same as well.

### Route choices across studies

Comparisons between the group types in the three studies demonstrate similarities in route planning choices. The top five routes planned across all studies are shown on the map in Fig. 3. Although most of the plans reported (71%) select one of these 5 popular routes, a visual of all unique route plans is shown in Fig. 7 to provide context for other proposed solutions across the three studies. In Table 4, we describe the same top five routes, rank-ordered by popularity of choice overall and for each study. In Table 5, we report indicators of route complexity for each of these top five routes. These indicators include distance, total number of turns on the route, and the number and proportion of turns that are unmarked (no visible street sign at or near the turn).

The indicators allow us to examine relative complexity for selected routes, relating complexity of a route to its popularity of choice; distance and fewest turns have commonly been reported as the most important indicators in peoples’ route selection criteria (for instance see Golledge, 1995). Despite participants being asked to minimize both time and distance, we find that distance of the route was not the sole criterion for decision-making. The fact that the shortest routes were *not* the most popular—alongside the evidence from the conversational analysis and think-aloud protocol analysis—suggests that participants recognized their uncertainty about successfully carrying out more complex route plans during situated navigation.

**Table 4:** Top five most popular route plans, by study

Route code	Ranked popularity	Prev. Study: Stranger Dyads	Study 1: Friend Dyads	Study 2: Solo Navigators	Total
A	1	12	6	13	31
B	2	7	5	6	18
C	3-4	4	5	3	12
D	3-4	7	4	1	12
E	5	4	5	0	9

**Table 5:** Route complexity measures for top five route plans

Route code	Ranked popularity	Distance (mi)	Total Turns	Unmarked turns (proportion)
A	1	0.52	3	0 (0%)
B	2	0.56	6	3 (50%)
C	3-4	0.36	4	1 (25%)
D	3-4	0.57	4	3 (75%)
E	5	0.27	6	3 (50%)

The most popular route, Route A (labeled on the map in Fig. 3), is indeed the least complex: It only includes three turns, with all turns marked with the relevant street sign in the environment, making them recognizable entirely by their street names. Out of all the plans reported across the studies, Route A would have the lowest cognitive load and therefore be easiest to remember. However, Route A is not the shortest of all possible routes, meaning that participants considered the greater simplicity of the route to be a worthwhile trade-off for traveling for a greater distance and time. Alternatively, the popularity of this route may serve as further support for the southern route preference hypothesis (Brunyé et al., 2012), but the study site used here does not provide enough control to allow us to affirm or deny this explanation.

In fact, although the planned Route E is the shortest in terms of measured distance on the map, it is impossible to carry out in the real-world environment, as there is no traversable path through the residential properties. Route C (0.36 miles) would therefore be the most efficient possible route from the origin to the destination point, which traverses the footpath but requires shortcutting through an open greenspace with no labeled pathway. There would have been high uncertainty by participants about whether this would be possible in the real-world navigation, which is corroborated by our review of the planning videos and verbal descriptions of routes. As shown in Table 4, only 12 dyads or solo navigators across the three studies reported this as their route plan.

The next most efficient route that was planned using the map and could be navigated in the environment was Route D (0.36 miles), which also uses the footpath labeled on the planning map. One primary barrier to selecting a route that took a footpath through the middle area appeared to be uncertainty about what was present in the ‘middle area’. In reality, this middle area (refer back to Fig. 1) is a vernal pool, which is a seasonally occurring pond or lake area that appears in times of high and/or sustained precipitation. In the map, this area was represented as a minimally shaded region with cross-hatching. It is crossed by a number of labeled footpaths on the provided map, so it is clear the participants may travel along those paths.

Future research in this area could systematically vary the types of turns involved among several routes to better elicit the relative contributions of a route’s features to a route’s complexity.

### Navigational performance

**Fig. 4:**
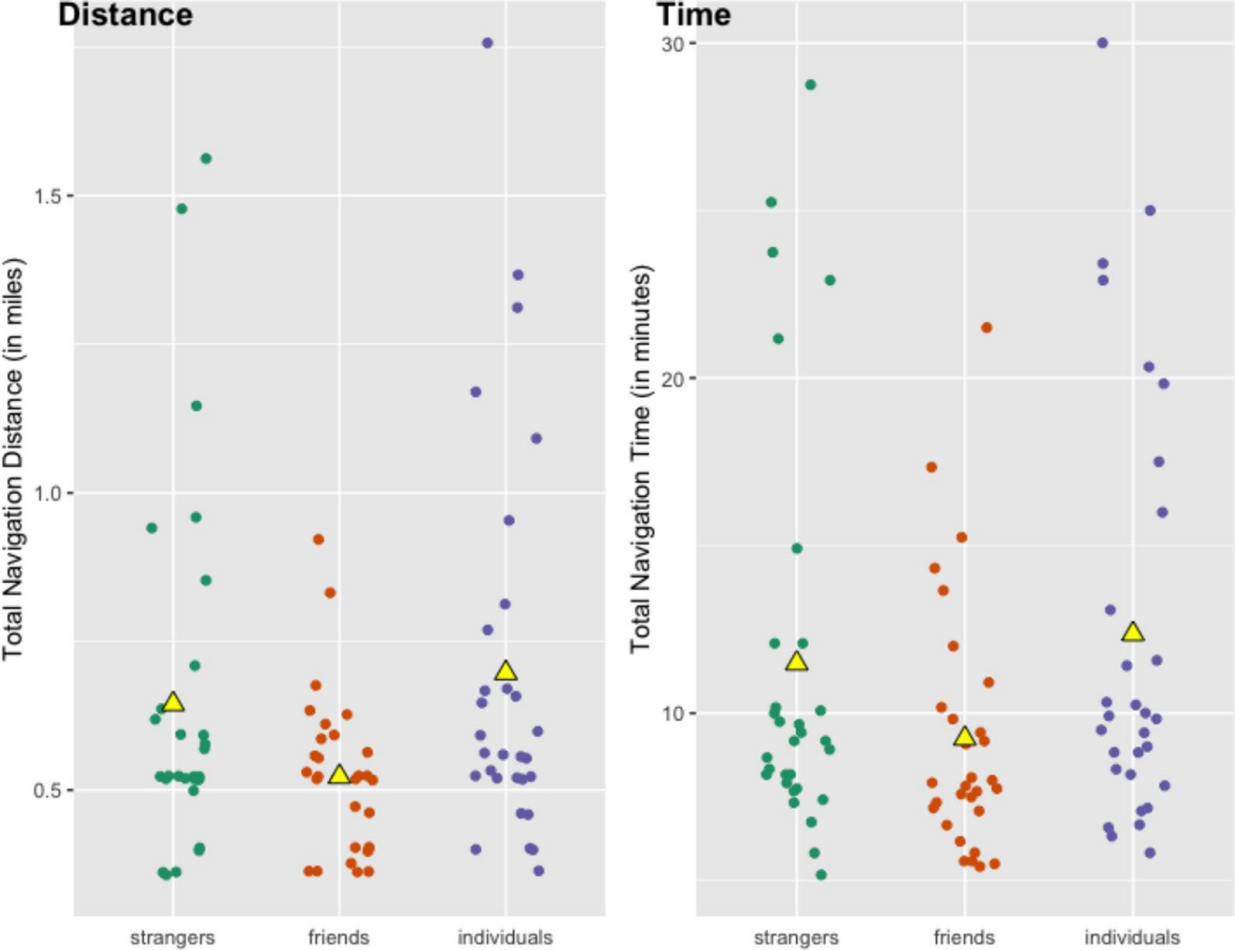
Jitter plot of total distance and time across the three studies. Triangle marker represents mean values

**Fig. 5:**
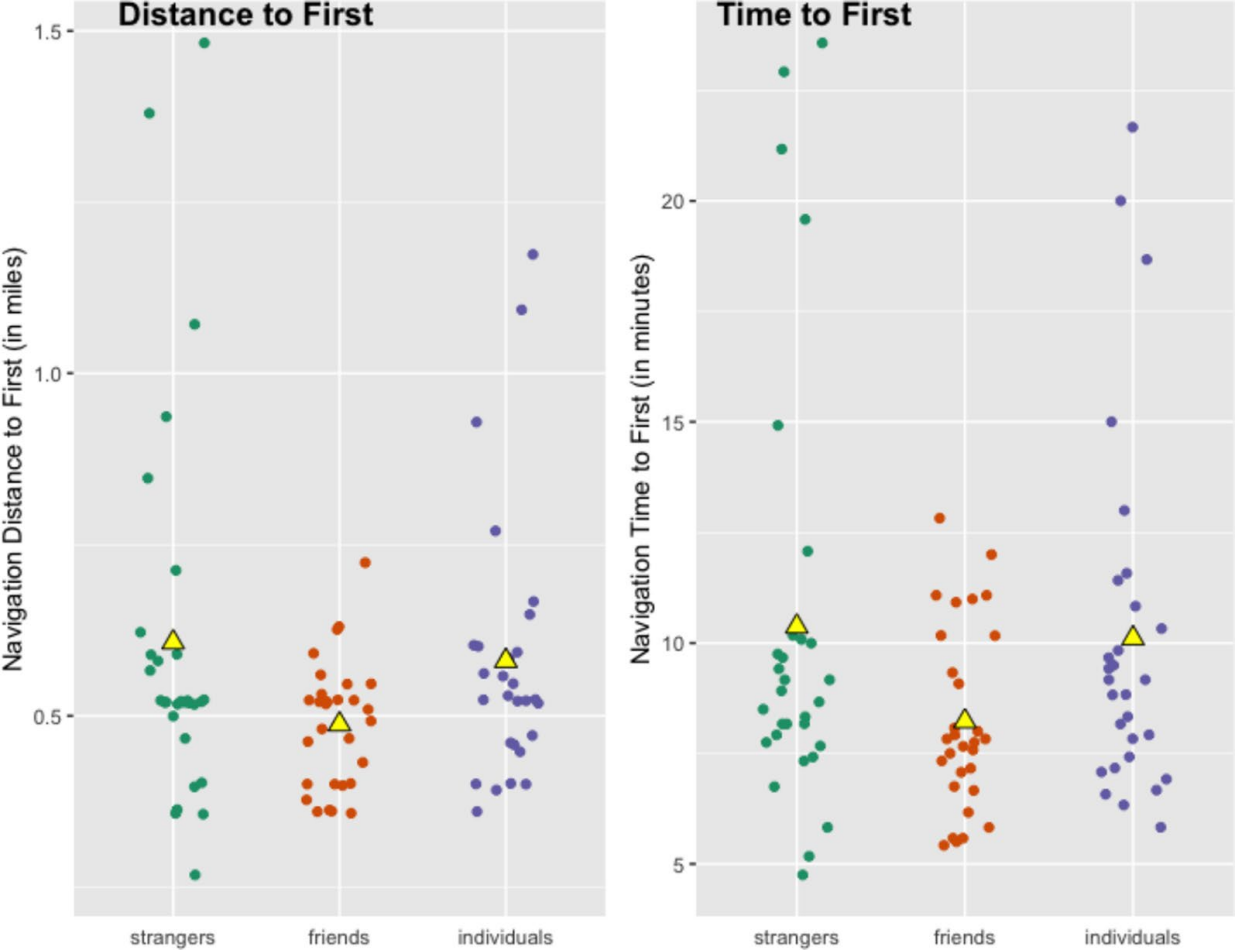
Jitter plot of distance and time to first attempted destination across the three studies

Table 6 compares the navigational performance of participants in dyads and as individuals across the three studies. Performance is reported as the success rate, average total time and time to first, and average total distance and distance to first attempt for participants across all three studies. See Figs. 4 and 5 for jitter plots of total distance and time measures for all participants across the three studies.

**Table 6:** Comparison of navigational performance across the three studies

	Success on navigation	Avg. time (total)	Avg. time to first	Avg. dist (total)	Avg. dist to first
Previous Study (Stranger Dyads)	26 of 30 (87%)	11′ 29″	10′ 22″	0.64 miles	0.61 miles
Study 1 (Friend Dyads)	22 of 30 (73%)	9′ 14″	8′ 14″	0.52 miles	0.49 miles
Study 2 (Solo Wayfinders)	16 of 30 (53%)	12′ 22″	10′ 07″	0.70 miles	0.58 miles

Due in part to the lower success rate of solo navigators as compared to dyads, total travel time and distance were also longer for individuals than in either of the dyad studies. There was also greater variability in individuals’ time and distance performance than in dyads’ performance.

Across the three studies, friend dyads demonstrated the most efficient travel in terms of both total time and total distance traveled, followed by stranger dyads, and lastly solo wayfinders (see Table 6). However, as can be observed in Fig. 4, these are likely due to outlier values that may impact these numerical comparisons. Overall, the participants with the highest range and standard deviation for total distance traveled were solo navigators (range 1.40 miles, *SD* = 0.33 miles), then strangers (range 1.20 miles, *SD* = 0.30 miles), whereas friend dyads showed the least variance (range 0.56 miles, *SD* = 0.13 miles) in total distance traveled. This same pattern held for total time traveled: there was the highest variability in values for solo navigators (range 24′ 10″, *SD* = 6′ 26″), followed by stranger dyads (range 23′ 35″, *SD* = 6′ 14″), and lastly friend dyads (range 16′ 05″, *SD* = 3′ 49″).

When assessing only those who were scored as successful in the navigation,^6^ we find that successful friend dyads were more efficient in their travel than successful stranger dyads and solo wayfinders. The 22 friend dyads who were successful navigated on average for a shorter time and distance to reach their destination (0.48 miles and 7′ 35″) than the 26 successful stranger dyads (0.58 miles and 9′ 48″) and the 16 successful solo wayfinders (0.51 miles and 8′ 16″). This means that of those who successfully reached the destination on their first attempt, it appears that stranger dyads took longer than friends and individuals and traveled further to do so.

Differences between the two metrics of navigational efficiency—distance and time—relate to planning behavior during navigation, attributable to time elapsed while planning in place (while movement is stopped). Solo navigators showed the highest correlation between total navigation time and distance, *r* = .97, *p* <.001, meaning they stopped moving very infrequently. We also found close relationships between time and distance during navigation for stranger dyads (*r* = .94) and friend dyads (*r* = .86). Differences between time spent and distance traveled during navigation can be attributed to pausing behavior, with the weaker relationship between time and distance indicating that friend dyads stopped to plan more often than planning while moving, as compared to solo wayfinders. We interpret this to be a result of the communicative needs between partners in a dyad, where members looked at each other, interacted via speech and gesture, and often simultaneously oriented their bodies in the same direction to mentally revisit their route plans or their ongoing progress while discussing their plans. Solo navigators did not “stop and plan” as often; we argue they also have less need to do so.

### Correspondence between planned and enacted routes

Similarly to the dyads in the previous studies, solo navigators traveled faster to their first attempted destination when following their planned route more closely. The high significant negative correlations between route overlap and time and distance to the first destination point to the same pattern across solo navigators, friend dyads, and stranger dyads. The relationship between adherence to the planned route and efficiency of navigation is consistently supported across the three studies, highlighting the closely-related role of planning to the act of efficient wayfinding through a novel environment.

**Fig. 6:**
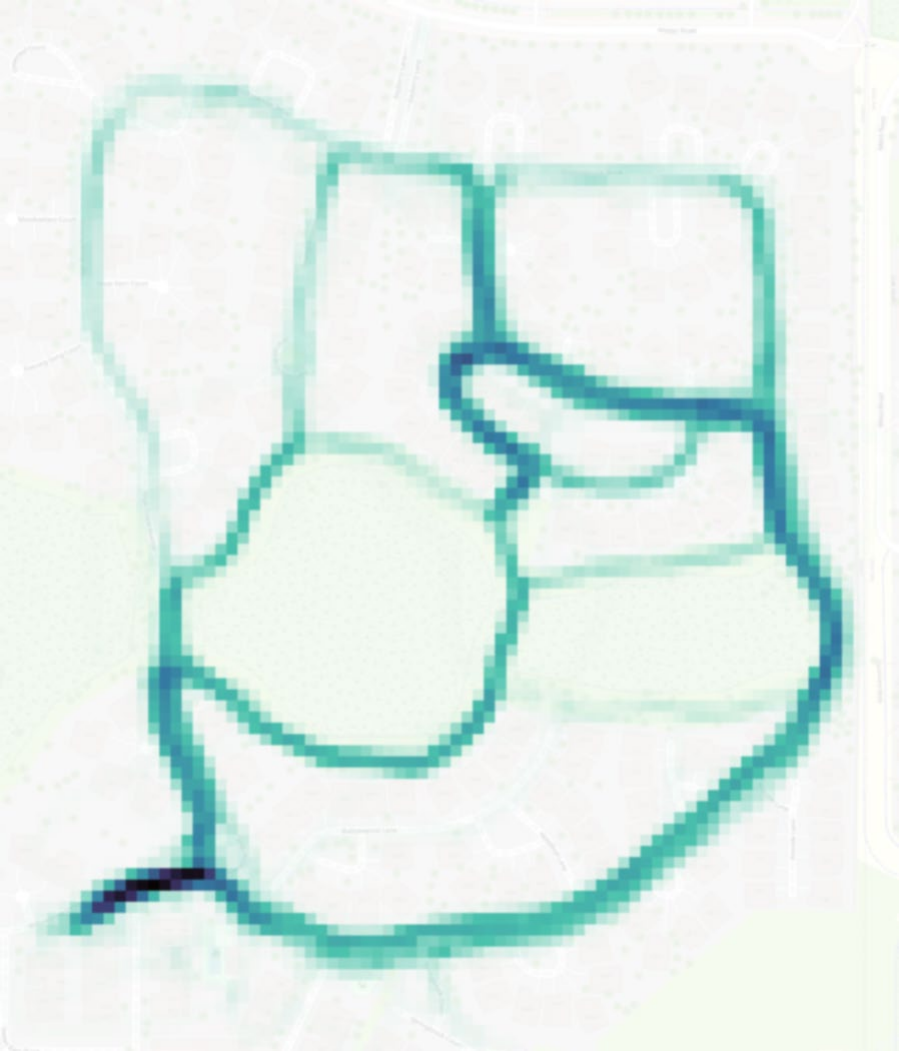
Heatmap of enacted routes across all three studies

**Fig. 7:**
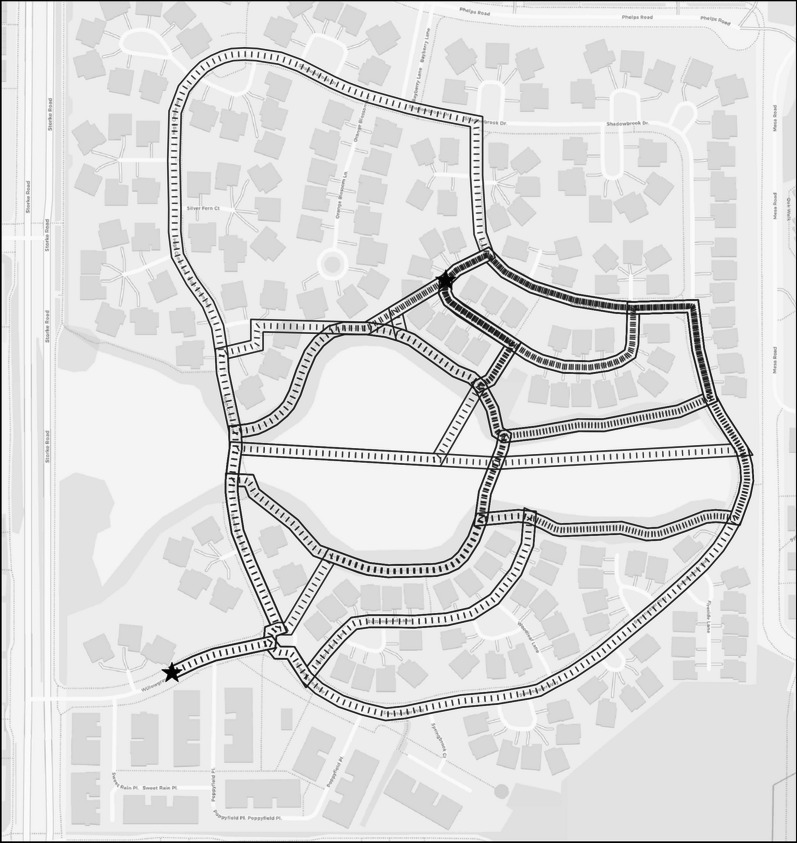
Map with overlaid representations of all of the unique route plans reported by participants across the three studies

The solo navigators’ distance ratio (1.55) was higher than that of stranger dyads (1.34) and of friend dyads (1.22). Therefore participants across all three studies walked more distance than originally planned, with solo navigators appearing to walk more extra distance during navigation despite similar lengths of planned routes across studies. A heatmap of enacted routes for all participants across the three studies is displayed in Fig. 6, showing that there was high variability in enacted navigational paths.

The average route overlap was also lowest for solo navigators (65.3%), followed by stranger dyads (69.1%), and highest for friend dyads (75.4%). The use of directly comparable groups in future work would help identify the statistical significance of these differences. As another look at the behavior of following original route plans, we found that only eight of 30 solo participants (8 of 30) followed their route exactly as planned. This is lower than the number of stranger dyads (10 of 30) and friend dyads (16 of 30) who did so, suggesting alongside the self-reported assessments that there were more issues by individuals in closely enacting planned routes when working alone.

## General discussion

In the two behavioral studies presented here, along with the previously published work in Bae and Montello (2019), we recruited participants to work in stranger dyads, in friend dyads, or solo to plan a route through a novel environment and carry it out in the real-world setting. Here we summarize the main findings across the three studies and their contributions to the field of spatial cognition and related research in social wayfinding (Dalton et al., 2019). Our findings show that this area of research holds much promise for examining the intricacies of individual and group spatial cognition, in terms of both the planning processes and the social interactional contributions to successful wayfinding.

In nearly all cases across solo participants, friend dyads, and stranger dyads, participants chose their route plan after considering others and were unlikely to have merely selected the first viable route they identified between the origin and destination. The most frequently planned routes across all solo participants and dyads shared in common that they were typically less complex despite being relatively longer. As planning time did not significantly differ between dyads and individuals, it is interesting to note that one person plans for about the same amount of time as two people, who must coordinate and agree upon a plan. However, planning time was not associated with navigational performance or success, indicating the greater importance of route choice and the enacted navigation.

One of the central questions from this study is whether individual wayfinders are more successful in their navigation than dyadic wayfinders. On nearly every metric of navigational performance measured, individuals performed more poorly than did dyads (whether strangers or friends). Overall, the navigational performance measures show us that individuals were less successful than both types of dyads, especially strangers, as fewer individuals reached the destination on their first attempt in the wayfinding task. Four solo participants gave up early on the task during the navigation phase, behavior which was not observed for either friend or stranger dyads. Social structure, even at the dyad level, seems to be an important motivator to persevere in the task at hand. This may manifest through increased persistence in the presence of a wayfinding partner or greater confidence in the dyadic or group-level abilities.

Differences in navigation efficiency as measured through travel distance or time were mostly not attributable to the measured sense of direction or personality characteristics. However, for individual wayfinders, higher Openness to New Experience was related to *less* efficient travel to the destination, suggesting more use of complex paths or more likelihood of exploration for those individuals despite uncertainty in the novel environment.

In terms of applied wayfinding strategies, we suggest that individuals faced more difficulty with the tasks of remembering and had fewer social resources available to them during navigation than did dyads. Much of this relates to differences in planning, decision-making, and memory during navigation. Individuals had a smaller pool of reported unique route plans and more often planned ‘safer’ or simpler but longer routes, hinting at lower confidence about successful navigation. Indeed, we showed that dyads were able to consult one another about their decision-making en route and sometimes recognized each other’s mistakes in time to correct them, which helped them follow their plans more accurately. Potentially, the social allowance for critique or argumentation over the route plans could lead to better reasoning (Mercier, 2016), and therefore better wayfinding performance. In contrast, when a solo navigator made a mistake in their progress such as forgetting a turn along the route, they had no one to remind them.

In the context of social wayfinding, individuals may have access to fewer collaborative resources than those in dyads and potentially larger groups, who have additional means for remembering and assessing alternatives. There were many complex tasks involved with situated navigation, including performing the correspondence between the remembered map and the physical environment, rehearsing the route plan and anticipating upcoming decisions, and flexibly adapting one’s route. Although individuals expressed their own uncertainty at points throughout navigation, not being socially accountable to a partner during navigation may have led to less questioning of their ongoing progress. This in turn can mean fewer ‘checks’ on their navigation or challenges to their decision-making, as the solo navigator remains entirely responsible for their own success.

### Open questions and future work

Our work demonstrates that the related roles of confidence, exploration, and risk-taking within dyads and larger social groups should be further investigated in the wayfinding context. For instance, how does social role-taking facilitate or inhibit spatial information sharing within the group? How does interaction among dyad members change over the course of a wayfinding episode, such as through the emergence of leadership within groups? How does gender impact social dynamics within dyads? Differences based on gender were inconclusive, due to the uneven distribution of gender pairings across dyads, but are worth considering in the design of future behavioral studies involving dyadic wayfinding.

With regard to issues such as navigational efficiency, we can ask questions about the role of pausing or stopping during travel. When is it more effective to stop and focus on planning versus continuing to plan while on-the-move? Although social relationships were not subdivided beyond “friends” and “strangers” across this set of studies, future work may find further levels of social group classification to be of interest. Additionally, because interpersonal interaction is necessarily shaped by cultural and societal influences, cultural differences may be a fruitful way to investigate group navigational behaviors.

Our studies provide an initial step toward future work in this area, which should further consider the impacts of social context as situated in real environments. We recognize the limited generalizability of this set of studies, in that it takes the form of a single navigation trial in only one physical environment. For that reason, future work should extend the study of situated social navigation in multiple other environments as well. There are many open questions in social wayfinding research that call for strong interdisciplinary approaches. Open areas of research include elucidating differences in information needs between individuals and groups, the classification of social groups with relevance to wayfinding and other spatial behaviors, and establishing how uncertainty is reduced or heightened when groups work together to navigate through a new environment.

## Conclusions

This research expands our understanding of wayfinding in a real-world situated context, referring to both the physical environment as well as the social environment of working jointly with a partner. The complexity of human behavior in groups calls for interdisciplinary methods of inquiry and approaches to understanding, such as we demonstrate. By bringing together the diverse methodologies and ways of knowing represented by geography, psychology, and sociology, we investigate wayfinding behavior in both a real physical and social context. In doing so, we outline the ways in which social behavior influences dyads’ planning of routes and the enactment of routes in the context of navigation through a novel environment. This has broader applications to general group decision-making research in various disciplines and can inform the design of navigational aids, such as signage and wayfinding systems, for both built and natural environments, including navigational support systems for use by multiple people.

Social wayfinding is a relatively recent area of inquiry emerging from the field of spatial cognition (Dalton et al., 2019) which presents opportunities for learning about joint attention, collaborative memory, and communication practices (Heft, 1996). Several important and complex processes of cognition are involved in carrying out a wayfinding task, including prospective planning, problem-solving and decision-making, and dealing with uncertainty, all of which are subject to social effects. However, wayfinding research thus far has largely focused on the individual scale of analysis, with the study of individuals finding their way through an environment alone.

In our presented studies, we find evidence to support important differences in spatial route planning and situated navigational behavior, all within the important socially-situated context of working either with a familiar or unfamiliar partner, or solo. Individual differences between participants, such as in sense of direction ability or personality attributes, is likely to influence how they show up in such social wayfinding situations. We argue that future work in the area of social wayfinding must consider the complexities of social interaction alongside specific spatial strategies employed in route planning and wayfinding. Taken together, these studies contribute research that is centrally focused on social aspects of wayfinding, and raises important new questions about the interplay between the peoples’ physical and social environments when engaged in navigation.

## Open practices statement

1. Navigational performance data for all participants is available upon request, including time and distance measures. Video-recordings are not available directly, due to issues surrounding personally identifiable information (PII) such as faces, names, and personal conversation. However, selected transcripts of conversation can be provided upon request with PII censored.
2. Experiments were not pre-registered.

### Supplementary Information

Supplementary file 1

## Data Availability

All video- and audio-recordings taken during data collection for this study are stored securely and password protected for use in analysis. The datasets generated and analyzed during the current study are not publicly available due to protection of personally identifiable material but generalized summaries are available from the corresponding author on reasonable request.

## Appendix 1: Comparison of individual difference measures

See Table 7.

**Table 7:** Comparison of individual difference measures across the studies

Measures	Prev. Study Averages	Study 1 Averages	Study 2 Averages	Differences across studies
SBSOD	3.9	4.0	3.9	*F*(2,87) = 0.16, *p* = .85
Extraversion	3.3	3.4	3.2	*F*(2,87) = 1.10, *p* = .34
Agreeableness	4.1	3.8	3.8	*F*(2,87) = 1.80, *p* = .17
Conscientiousness	3.6	3.4	3.7	*F*(2,87) = 2.34, *p* = .10
Neuroticism	2.8	3.0	2.8	*F*(2,87) = 1.49, *p* = .23
Openness	3.5	3.4	3.5	*F*(2,87) = 0.44, *p* = .65

## Appendix 2: Overlay of all routes planned by participants across the three studies

See Fig. 7.

## Footnotes

1 Participants completed the study in exchange for extra credit in an introductory level Geography course. Most participants were not Geography majors, however, so we did not expect them to have specialized expertise in the subject matter.

2 The selected friend did not need to be currently enrolled in a Geography class (and 9 were not).

3 The previous study reported in Bae and Montello (2019) involved only 2 male-male pairs, as opposed to 15 female–female and 13 female-male pairs.

4 Following a modified version of basic conventions in Conversation Analysis, adapted from the guide by Sacks et al. (1974), the transcription attempts to directly capture speech as produced rather than along orthographic rules, aligns overlapping speech between two speakers [within brackets], capitalizes louder speech, surrounds softer speech with *asterisks*, and represents upward inflections with ^^^. Gestures are described within ((double parentheses)). Pauses lasting less than a tenth of a second are represented as (.); longer pauses are shown with the duration in tenths of a second in parentheses.

5 Again, the “first attempt” is the first location where the participant reported that they had reached the destination, whether correct or incorrect.

6 Again, scoring of success was based on correct identification of the destination on their first attempt.
