# Supplementary material for: Wayfinding in pairs: comparing the planning and navigation performance of dyads and individuals in a real-world environment
Source: Cogn Res Princ Implic. 2024 Jun 21;9:40. doi: 10.1186/s41235-024-00563-9 (PMC11189867; doi:10.1186/s41235-024-00563-9)
Supplement: Supplementary file 1 [file 41235_2024_563_MOESM1_ESM.pdf]

Post-Navigation Survey

During navigation in the environment with your partner…

1. Did you and/or your partner take a path that was different from your planned route in any way? Describe if so.

2. Did anything unexpected happen (related to your navigation) while you and your partner were walking along your route? If so, what was unexpected?

3. Who acted more as the navigational leader? (circle one)
    1. I was leading more
    2. my partner was leading more
    3. neither was clearly leading more

4. Were there any points during which you felt lost or unsure about the route? If so, describe.

5. At any point did you and your partner disagree about the way to go? If so, describe.

6. Not considering how well you or your partner found the destination today, how confident are you in your partner's general sense of direction or navigation ability?
    a. very confident – I would never doubt their ability to find their way
    b. confident – I generally trust them to know where they're going
    c. average – I think they are about the same as most people in terms of navigation
    d. not confident – I would feel better if someone else were in charge of navigating

7. How long have you known your partner? (in months or years)
